# Endoscopist-related factors contributing to high-quality colonoscopy: results of a Delphi survey

**DOI:** 10.1007/s40037-013-0099-3

**Published:** 2013-12-05

**Authors:** Vivian E. Ekkelenkamp, Arjun D. Koch, Jelle Haringsma, Ernst J. Kuipers, Robert A. De Man

**Affiliations:** Department of Gastroenterology and Hepatology, Erasmus MC – University Medical Center, Room Ca-425, PO Box 2040, 3000 CA Rotterdam, the Netherlands

**Keywords:** Assessment, Gastrointestinal endoscopist, Delphi, Training

## Abstract

Education and competency assessment in gastrointestinal endoscopy is important. Concerning colonoscopy, it is not completely clear what the best way is to learn this procedure, what defines competency in colonoscopy, and which factors define a high-quality colonoscopy. The aim of this study was to determine the endoscopist-related factors that define a high-quality colonoscopy. A three-round Delphi survey among expert endoscopists was carried out. In round 1, the panel was invited to identify factors essential for a good colonoscopy. The listed factors were to be ranked during the second round. In the third round, a 5-point Likert scale was added. A reference panel was invited to assess the items as well. 14 expert endoscopists from the Netherlands were invited, of whom eight participated (57 %). A list of 30 items important for colonoscopy was formulated. After the following rounds, consensus was reached on 16 items. Validation was conducted among eight trainees and eight experienced endoscopists (response 100 %). The groups agreed on the importance of all but one factor (*p* = 0.001). This Delphi survey has made explicit the endoscopist-related factors that are important for optimal colonoscopy. This might provide trainers more support regarding concrete competency assessment of trainees in endoscopy.

## Introduction

Nowadays, colonoscopy is the most commonly performed gastrointestinal endoscopic procedure in Western countries. The implementation of colorectal cancer screening programmes all over the world has largely increased colonoscopy demand, and put major emphasis on quality assessment [1, 2]. Indicators such as cecal intubation rate (CIR) and adenoma detection rate (ADR) are now recognized worldwide as outcome parameters on quality [3]. However, these outcome parameters primarily indicate quality over larger numbers of procedures and do not address the quality of a single colonoscopy. Quality is narrowly linked with competency, which is endoscopist-dependent.

The ultimate goal of education and training in colonoscopy is to deliver competent endoscopists, but concrete measures to define competency in colonoscopy are sparse. Procedural knowledge and skill development are both important domains in endoscopic education and determining competency should be based on at least parts of those domains. Skills acquisition in colonoscopy is a topic that has increasingly received attention [4–7]. There are nonetheless no standards available for structured assessment of trainees and it is not clear which domains deserve the most attention. Assessment forms that are commonly used in Dutch practice are the objective structured assessment of technical skills (OSATS) form, similar to the direct observation of procedural skills (DOPS) form developed in the UK and the Rotterdam Assessment Form for colonoscopy (RAF-c) [4, 8]. The RAF-c is primarily developed for self-assessment of colonoscopy and therefore not appropriate for expert assessment of the trainee’s competency. The OSATS is being used in different specialties and directly derived from the surgical variant. Even for surgical procedures, the value of OSATS for measuring progress as well as defining competency is not clear [9], let alone for colonoscopy. Therefore, colonoscopy trainers as well as trainees feel that the OSATS and RAF-c do not reflect all aspects of colonoscopy training and should be optimized.

There is thus a need to identify specific factors that can be used in the assessment of colonoscopy quality and in skills assessment of trainees in colonoscopy. There are different methods to explore the opinion of experts on this topic. A Delphi survey is a well-recognized method to reach expert consensus through an anonymous group process [10–14]. The aim of this study was to visualize an optimal colonoscopy through the eyes of expert endoscopists, with explicit identification of important factors that define a high-quality colonoscopy.

## Methods

A three-round Delphi survey among expert endoscopists in the Netherlands was conducted. Endoscopists were selected based on their experience in colonoscopy and reputation in the field. Invitations to participate in the survey were sent to the selected endoscopists by e-mail and regular mail. Round one of the survey was sent together with the invitation. This was an open round. The panel was invited to openly list endoscopist-related factors that were in their opinion essential for a high-quality colonoscopy. The factors identified in the first round were collated and compiled into a new questionnaire.

This list was distributed as the second round of the Delphi study. Again, the questionnaire was spread by e-mail and regular mail. In round two, the listed factors had to be ranked by the expert panel. The most important item was ranked as no. 1, the second most important item as no. 2, and so forth. The outcome of the ranking in round two was sent back to the panellists as feedback.

The third round consisted of a consensus round. Now, a five-point Likert scale with values ranging from ‘1 = not important’ to ‘5 = very important’ was added to each item. After the three rounds of this Delphi survey were completed, two reference panels of trainees and certified endoscopists were put together. They were invited to assess the items that resulted from this Delphi study on the same five-point Likert scale. The purpose of this last evaluation was to assess whether the results could be extrapolated into clinical practice as items for assessing competency.

**Fig. 1:**
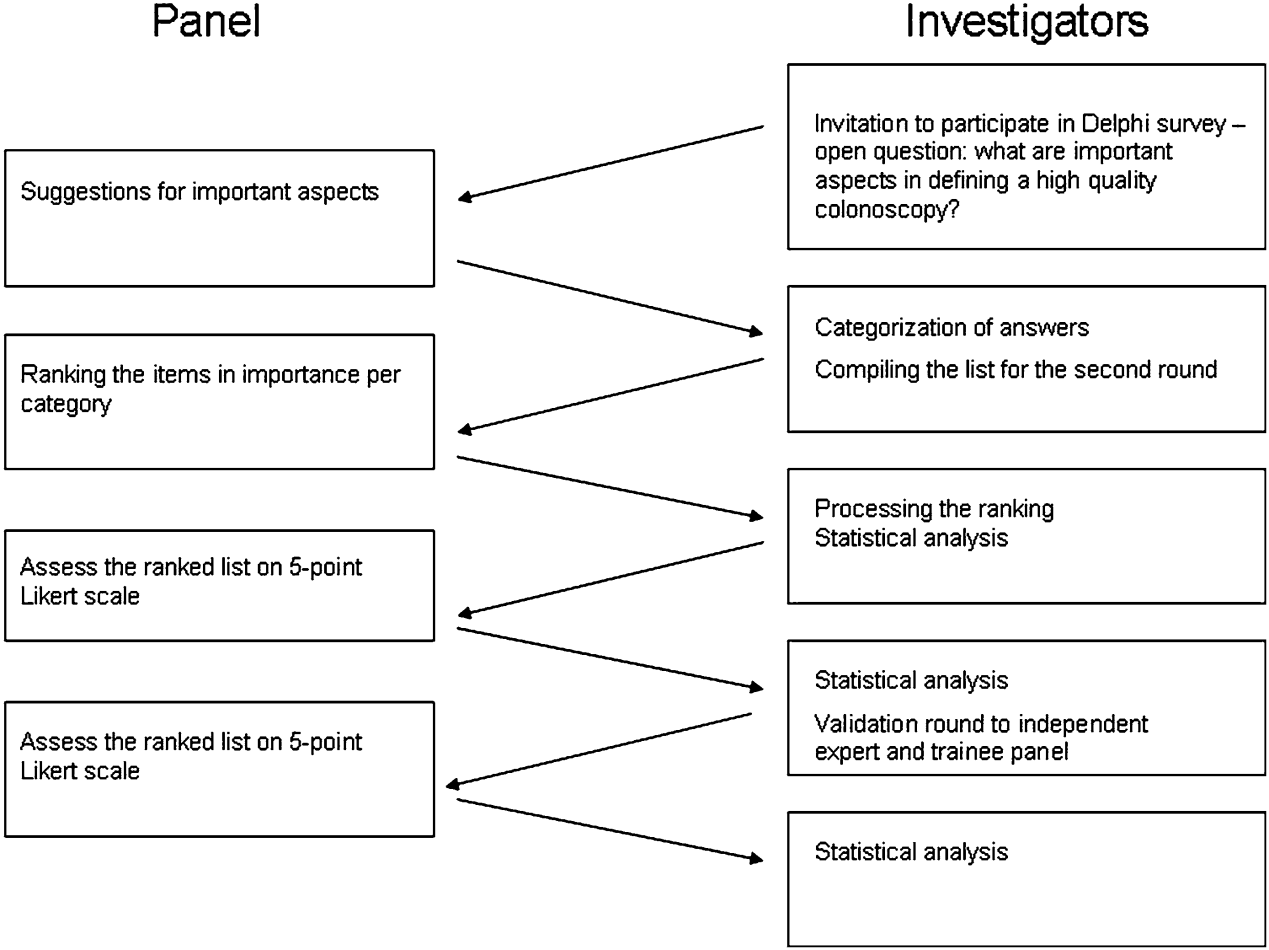
Flowchart of the Delphi process

Figure 1 shows the flowchart of the Delphi process as it was carried out in this study.

### Data analysis

Mean scores per item were calculated after the second round and mean Likert scores were calculated after the third and validation round. Cronbach’s alpha was used to quantify the reliability of the summation of entities, in this case the members of the Delphi panel. Where the responses of the members are highly correlated, they are considered to be internally consistent or homogeneous. A value of >0.8 was considered significant for consensus. Kendall’s tau-c was used to analyze the scores of the validation panels. A *p* value of <0.05 was considered significant. Analysis was carried out using IBM SPSS Statistics 20.

## Results

### Expert panel

Initially, 14 experienced endoscopists were identified and invited to participate in the Delphi study. They were selected based on their reputation in the field and they had to have a colonoscopy experience of >1,000 procedures. In the first round, 10 out of 14 endoscopists responded (71 %). Those 10 experts were invited for the second round; 9 out of 10 responded (90 %). Eight out of nine experts (89 %) responded to the third round. The overall response rate was 57 %.

### Round 1

The first questionnaire resulted in an overall list of 30 items: 10 in the first category representing efficacy and endpoints and 20 in the category representing safety and behaviour. Table 1 lists the factors provided by the panel. Some items were mentioned by different panel members, but these were only entered once in Table 1.

**Table 1:** Results of the first and second round of the Delphi survey

Efficacy/endpoints: mean score		Safety/side effects/behaviour: mean score	
Knowledge			
Adequate identification of endoscopic image	3.6	Knowledge of own boundaries	2.4
Basic colonoscopy technique	4.2	Knowledge of material and options for polypectomy	4.2
Knowledge of complications and registration	4.9	Knowledge of the burden for patients	4.4
Use of ADR as marker of performance	5.8	Understanding and solving loops	4.4
Knowledge of anatomy	8	Knowledge of calmly withdrawing the scope	6.6
		Use of CO2 insufflation	7.9
Skills			
Cecal intubation rate	4.2	Skills and hand-eye coordination	3.1
Polyp detection and removal	7.8	Rotation and straightening of the scope	4.7
Competency in intervention techniques	8	Patience and precision	6.1
Duration of the procedure and withdrawal time	8.6	Endoscopy with clear view	6.1
Proper assessment of mucosa	9	Small, gentle movements	6.1
		Anticipation and tip control	7.1
		Minimizing insufflation	7.9
		Proper position for intervention	8.8
		Feeling of equipment	8.9
		Adequate and ergonomic placing of equipment	9.6
		Localizing optimal pressure points and effects	9.6
		Scope positioning by changing patients position	9.9
		Experience of patients	10
		Use of opioids	10

### Round 2

In the second round, the random list with factors was sent to the expert panel members who had completed the first round. They were asked to give a number to each item in order to rank the importance. Items with number 1 were considered to be the most important and items with the lowest numbers the least significant by the expert panel. Cronbach’s alpha was 0.94, indicating a high level of agreement between the panellists. After analyzing the ranking of the different items, the list was segmented in order to provide a clear overview of the items. Two subclassifications were created for both categories, i.e. ‘knowledge’ and ‘skills’. Items were divided in those classifications by the investigators. The results are also shown in Table 1.

### Round 3

In the third round, the list as described in Table 1 was presented to the expert panel with an additional Likert scale. The mean score per item on the Likert scale, with a minimum of 1 and a maximum of 5, varied from 3.1 to 4.9. Cronbach’s alpha was 0.60 calculated over all items.

After this analysis, a list of items with the highest scores on the Likert scale per category was created. For each score of Likert 4 or 5, items received 1 point. These points were added up and the top three items with the highest scores per category were selected. When more than three items had the same amount of points, all of them were selected. A new list of factors important for a high-quality colonoscopy was created. All items in this selection, except for ‘assessment of mucosa’ (*n* = 1), received a score of 4 or 5, representing the values important and very important.

### Validation

Two validation panels were created. One panel consisted of eight experienced endoscopists working in our endoscopy department; the other panel was composed of eight gastroenterologists in training. We created these validation panels in order to evaluate the feasibility of using these items for assessing competency in clinical practice. All sixteen selected panel members returned the survey.

Cronbach’s alpha was calculated for all three panels together. This was 0.86, which means that a high level of consensus was reached. To explore the differences in scores of the three groups in more detail, Kendall’s tau-c was calculated. There were no significant differences in scores between the three groups for all items, except for ‘proper position for intervention’ (Kendall’s tau-c = −0.41; *p* = 0.001). Further evaluation showed that trainees scored this item significantly lower than the expert panel (*p* = 0.001). Table 2 shows the final result of this Delphi survey.

**Table 2:** Final results of Delphi survey

Efficacy/endpoints	Safety/side effects/behaviour
Knowledge	
Adequate identification of endoscopic image	Knowledge of own boundaries
Basic colonoscopy technique	Knowledge of material and options for polypectomy
Knowledge of complications and registration	Understanding and solving loops
Skills	
Cecal intubation rate	Skills and hand-eye coordination
Polyp detection and removal	Patience and precision
Competency in intervention techniques	Small, gentle movements
Assessment of mucosa	Minimizing insufflation
	Proper position for intervention
	Feeling of equipment

## Discussion

This Delphi survey has made explicit the endoscopist-related factors that play an important role in defining an optimal colonoscopy. Experts reached consensus on this topic in a three round survey. A list of 16 items was identified during the process. A validation panel of endoscopists and trainees agreed on the importance of nearly all the factors. This may provide trainers more support regarding concrete competency assessment of trainees in endoscopy.

It is important to assure quality in endoscopy training, but to be able to do that, a standard needs to be set [15]. Up until now, there is no universal method to assess a trainee’s ability and capacity. There have been several studies published on skill development in colonoscopy in general, not focusing on assessment. These studies mainly addressed motor or technical skills, such as CIR [4, 16]. In one study, a learning curve for CIR was created through self-assessment, which was a novel method to gain insight into progression of learning [4]. However, objective assessment by a supervisor did not play a role in this study. In order to provide a more complete picture of competency, objective assessment of other factors next to CIR is necessary. When taking a closer look at the DOPS evaluation method [17], the focus again lies mainly on technical skills. The Mayo Colonoscopy Skills Assessment Tool (MCSAT) is an assessment device as well, developed to assess gastroenterologists in training on their colonoscopy performance [18]. There are quite some similarities between the results of this Delphi study and the DOPS and MCSAT assessment forms. Basic colonoscopy technique, suction and loop recognition and reduction can be found in each of the tools. The same goes for adequate visualization of the mucosa and identification of landmarks and pathology, as well as completion of the procedure (CIR) and applying the correct intervention.

However, there were definitely some new aspects identified through our Delphi study. Where MCSAT and DOPS really focus on technical skills, as mentioned before, our experts valued factors such as knowledge of own boundaries, patience and precision and knowledge of complications very highly. The MCSAT mentions cognitive skills only once and does not specify them. Knowledge of material and equipment was also considered important by our experts; this is not mentioned at all in both the existing assessment forms. In summary, the existing assessment instruments are imperfect with comparable aspects regarding technical skills, but the Delphi study added an important area for assessment with items on knowledge, safety and behaviour.

The MCSAT was primarily based on literature and guidelines. A focus group of experienced endoscopists from one centre (Mayo Clinic) reviewed the blueprint, which eventually resulted in the final form. In this process, there is a risk that endoscopists with strong opinions have great influence on the final composition of the assessment form. A Delphi process seems more likely to result in honest answers and therefore a better representation of the complete panel’s opinion. Nonetheless, there are similarities between the forms despite the different methods of developing them, i.e. pre-procedural assessment, safe advancement of the scope and adequate visualization of the mucosa. The Canadian guidelines on endoscopic quality indicators were put together through a Delphi approach as well [19].

In this study, a Delphi survey was used to determine specific factors in defining a high-quality colonoscopy. The Delphi process is a well-recognized method to achieve consensus in a group on a given topic. It is a commonly used approach and the method utilizes the information from experience and knowledge of the panel members, mainly experts. Delphi has an anonymous nature. This could be an advantage because there is no place for dominance of specific panel members. On the other hand, discussions and hearing other’s arguments might lead to a higher level of consensus, because learning about different perspectives could influence the opinion of panel members.

The final list of items identified by the expert panel was sent to two validation panels, consisting of certified endoscopists and trainees in gastroenterology. Those panels agreed on the importance of almost the entire list; only one item was not assessed as important by the trainees as by the expert panel. The outcome of this internal validation makes it more likely that the factors can be extrapolated to clinical practice and are indeed important for assessment.

One limitation of this study is that the number of participants in the expert panel was relatively small (overall, eight experts completed the survey). There is, however, no strict guideline for the number of panel members when carrying out a Delphi study. The loss of two panellists during the second and third round is another limitation. The responses of those two members could have influenced the outcome and agreement, especially when taking into account that the overall number of panellists was relatively small.

This Delphi study was carried out in order to gain insight into the thoughts of experts on competency in colonoscopy and eventually to create a clear list with items on which competency can be based. The first round was open and no suggestions were given. This resulted in an unbiased response of the panel members, since they had to define for themselves what factors they considered important. After the second round, a high level of agreement was reached; after three rounds, Cronbach’s alpha was slightly lower. In other words, there was a high agreement on the overall set of items, but importance scores on the Likert scale per separate item varied. The first evaluation of the items (after round two) was carried out through a simple ranking; in the second assessment, a Likert scale was added. After evaluating the results of the scores on this Likert scale in the last round, a selection was made of the items with the highest mean scores. After this selection, expert agreement on this last group of factors was again higher. It can therefore be concluded that the final record of factors important in defining competency in colonoscopy is correct, given that Cronbach’s alpha had increased again, compared with the previous analysis. After evaluation of this list by the validation panels, consensus was reached on all items but one. Compared with the expert panel and the certified endoscopists, the trainees considered ‘Proper position for intervention’ less important. One possible explanation for this is that trainees have less experience in intervention techniques and still lack knowledge in this area.

The next step in this research process will include the development of a new assessment form with the factors derived from this Delphi study and to test and implement it in clinical practice.

## Conclusion

This Delphi study provides valuable insight into the opinion of experts regarding competency in colonoscopy. Endoscopist-related factors for a high-quality colonoscopy have been made explicit through this survey. Taking the outcome into account, we believe that it is justified to implement the factors that resulted from this study in an assessment device for trainees in colonoscopy. This might provide endoscopy trainers more support in concrete competency assessment.

## Essentials

- Assessment of performance in colonoscopy training is important.
- Aspects of knowledge should also be addressed in assessment, in addition to assessment of technical skills.
- The endoscopist-related factors identified in this study should be implemented in an assessment device for colonoscopy trainees.
